# Alterations in Energy Partitioning and Methane Emissions in Murciano-Granadina Goats Fed Orange Leaves and Rice Straw as a Replacement for Beet Pulp and Barley Straw

**DOI:** 10.3390/ani11010038

**Published:** 2020-12-27

**Authors:** Tamara Romero, José L. Palomares, Vicente J. Moya, Juan J. Loor, Carlos Fernández

**Affiliations:** 1 Instituto de Ciencia y Tecnología Animal, Universitat Politècnica de València, 46022 Valencia, Spain; tarorue@upvnet.upv.es (T.R.); jopalom@dca.upv.es (J.L.P.); vjmoya@dca.upv.es (V.J.M.); 2 Department of Animal Sciences, Division of Nutritional Sciences, University of Illinois, Urbana, IL 61801, USA; jloor@illinois.edu

**Keywords:** orange leaves, rice straw, methane emissions, goats, maintenance

## Abstract

**Simple Summary:**

Reducing methane emissions in ruminants with the recycling of agro-industrial by-products is of great importance today. Pruning waste from citrus trees is currently burned or incorporated into soil. Regarding rice straw, this waste is traditionally eliminated through controlled burning, releasing into the atmosphere large amounts of greenhouse gases as well. The aim of this study was to convert this recovered waste into a new animal feed capable of reducing methane emissions in ruminants. The interest in use waste by-products for ruminant nutrition is increasing. Therefore, we replace the beet pulp and cereal straw from dry-non-pregnant goats’ diet with orange leaves and rice straw with the objective of studying their effect upon intake, digestibility, energy efficiency, carbon and nitrogen balance, and methane emissions.

**Abstract:**

Considering the huge quantities of crops by-products and pruning waste such as rice straw and citrus leaves produced annually worldwide, and their potential pollution capacity, recycling as feed for livestock is an alternative. The objective was to study these by-products effect on energy balance and methane emissions in 10 Murciano-Granadina goats at maintenance. The control diet (CTR) included barley straw and beet pulp while the experimental diet (ORG) consisted of rice straw and orange leaves. Differences were found for energy intake (248 kJ/kg of BW^0.75^ greater for CTR than ORG). The intake of metabolizable energy was 199 kJ/kg of BW^0.75^ lower in ORG than CTR, and the energy efficiency was higher with CTR (0.61) than ORG (0.48). Protein retained in the body was 9 g/goat greater with CTR than ORG, and fat retention in the body was approximately 108 g/goat greater with CTR than ORG. Despite more unfavorable energy balance in response to feeding ORG than CTR, the retention of body energy was always positive. Reductions in CH_4_ emissions were detected when goats were fed ORG diet (from 22.3 to 20.0 g/d). Overall results suggested that feeding orange leaves and rice straw was effective in reducing CH_4_ emissions without adversely affecting energy balance.

## 1. Introduction

Ecological leftovers such as crop residues, food wastes, and agro-industrial by-products constitute human-inedible feed biomass [1]. Huge quantities of waste biomass are generated as agricultural and food industry by-products, explaining around 30% of global agricultural production [2]. Usage of such “less food-competing” biomass as feedstuff in animal diets is a potential approach to diminish food feed competition that can also help mitigate environmental effects of livestock.

The combination of livestock with crop production is a manner of creating sustainable farming systems that purpose to optimize resource usage. Crop residues and fibrous agro-industrial by-products do not enclose the nutrient balance needed to support efficient ruminal fermentation and animal performance. In countries with more specialized livestock systems, rice straw is considered of low nutritional value and is burned, however in developing countries where livestock are integrated with cropping, it is a valuable resource [3]. Despite this, in many areas such as Southeast Asia and Mediterranean countries, cereal straw is used as fodder providing fiber.

Rice (488 million tons of husked rice) is the world’s third major cereal crop after corn and wheat but produces the greatest amount of crop residues in the form of rice straw [4,5]. For instance, Spain produces 525,504 tons/year of rice straw [6]. Other important horticultural by-products in Spain come from citrus trees. Spain is one of the most important citrus production regions in the world and is acclaimed for cultivation of oranges and mandarins. Consistent with [7], Spain generates 1.87 million tons per year of pruning waste (on dry matter basis), of which almost 500 g/kg is leaves and 500 g/kg wood [7].

The main limitation of rice straw and orange leaves as feedstuff is the low nitrogen content and digestibility; these by-products are composed of cell wall constituents with slight soluble cell components, thus have to be degraded via microbial fermentation. The productivity of animals fed these waste by-products could increase with supplementation with a source of protein or N. Thus, these crops and pruning wastes could be converted into a valuable feed product for ruminants [3,8]. Furthermore, due to its high essential oils content, orange leaves could be advantageous for reducing methane productions from ruminants [9]. 

Studies carried out in livestock are scarce. With the use of citrus pulp, there are many studies, but not with citrus leaves, and as an example we can point to some work in broilers [10] and goats [11]. With rice straw there are more studies, mainly from Southeast Asia [12,13,14]. However, there are no studies that combine citrus leaf and rice straw in the same ration.

Considering the large amount of rice straw and citrus leaves produced annually worldwide, and their potential pollution capacity, especially when burned, revalorization and reutilization of these by-products as a complementary feed sources for livestock is a relevant subject in the exploration of the circular economy. Thus, the objective of this study was to replace beet pulp and cereal straw with orange leaves and rice straw in the diet to fed dry-non-pregnant goats with the aim of studying their effect upon intake, digestibility, energy efficiency, carbon (C) and nitrogen (N) balance, and CH_4_ emissions.

## 2. Materials and Methods

### 2.1. Ethics Statement

Experimental procedures were approved (with reference 2017/VSC/PEA/00182) by the Animal Use and Care Committee of the Universitat Politècnica de València (UPV, Valencia, Spain). Besides, the procedures followed the practice codes for animals in experimental work advised by [15]. 

### 2.2. Animals and Diets

The experiment was managed at the Experimental Farm belonging to the UPV Animal Science Department (UPV), Valencia (Spain). Ten dry, non-pregnant multiparous Murciano-Granadina goats were chosen and assigned to two homogenous groups; five goats per group based on alike body weight (BW; 44.1 ± 3.65 kg) in a cross-over design (2 treatments crossed with 2 periods). The breed used was the Murciano-Granadina as it is a typical native breed of this geographical area of Spain. Females have been chosen because the production system for this breed revolves around milk production and the mothers are in standardized lactations at 210 days. During the drying phase it is important to have a maintenance diet for the animals, and ideally it is to be able to use crop residues and fibrous agro-industrial by-products. Treatments involved two different pelleted diets (Table 1). The control diet (CTR) included barley straw and beet pulp and the experimental diet (ORG) consisted of rice straw and orange leaves. Both diets included sunflower meal as protein source and sugarcane molasses to get a good pellet texture. Diets followed nutrient recommendations by Calsamiglia et al. [16] for goats at maintenance. The feed offered was 2 kg, which was distributed twice a day; at 0900 h and at 1600 h. Water and mineral vitamin blocks were free for all goats.

### 2.3. Experimental Procedure and Sampling

Apparent total-tract digestibility, partition of energy, balance of C and N, and CH_4_ emission were determined. The crossover design experiment consisted of two 38-day periods and at the beginning and end of the experimental period the BW were obtained (Tru-Test FastWeight Crate, Datamars Livestock, AFB Farm supplies, Cheshire, UK). Goats were allocated in pens and fed the experimental diets for 14 days during adaptation, then allocated for an additional 7 days to individual metabolism crates at thermoneutrality (20–23 ºC; Hobo probe, ONSET data loggers, Cape Cod, MA, USA). During the next 5 consecutive days, daily feed offered, orts, and total fecal and urine output were recorded and collected for each animal. Plastic buckets for urine collection contained 100 mL 10% (*v*/*v*) of H_2_SO_4_. Representative samples (10%) of diet, feces, and urine were stored at −20 °C and pooled for chemical analysis. On the last day of the digestibility trial and before the morning feeding, ruminal liquor samples were sampled using a stomach tube and pH was immediately determined (portable pH meter Model 265A, Orion Research Inc., Beverly, MA, USA). A ruminal liquor sub-sample was acidified with 50% H_2_SO_4_ and frozen until ammonia-N (NH_3_-N) determination. The other ruminal liquor sub-sample was mixed with H_3_PO_4_ and kept frozen until volatile fatty acids (VFA) analysis. Jugular blood was sampled (10 mL tubes treated with EDTA) and centrifuged for plasma separation and then stored at −20 °C. Afterward, goats were moved for 2 days to pens prior to gas exchange procedures.

Gas exchange determination (oxygen: O_2_; carbon dioxide: CO_2_ and methane: CH_4_) was quantified for each goat during a 24 h period. The system used for gas exchange quantification was based on an indirect calorimetric system designed for small ruminants. The respirometry system was armed with 2 ventilated head-hoods, 2 flow-meters (Thermal Mass Flowmeter Sensyflow VT-S, ABB, Alzenau, Germany), and 2 air suctions afforded by centrifugal fans (CST60 Soler Palau Inc., Parets del Vallès, Barcelona, Spain). Gas analyzer measured O_2_ following paramagnetic principle, and CH_4_ and CO_2_ were measured following the infrared principle (Easyflow Gas Analyzer, model 3020, ABB, Alzenau, Germany). Reference gases were used to calibrate the gas analyzers before each test, and for a deep and detailed explanation of the portable open-circuit respirometry system used in this study it is advisable to consult the following works: Fernández et al. [17,18,19]. Further, the entire system was gravimetrically calibrated by injecting pure gas N_2_ and CO_2_ into the head-hood [20] (precision scale MOBBA mini-SP 0.2–30 kg, Industrial Weighing System, Barcelona, Spain). With the calibration of the entire system, the calibration factors were obtained according to Brockway et al. [21]. The O_2_ consumption and the CH_4_ and CO_2_ production were calculated as described by Aguilera [22]. Atmospheric air sample (the blank for calculations) was sampled and analyzed before and after each determination made in goats.

### 2.4. Chemical Analysis

Feed, feed refusals, and fecal samples were first dried in a forced-air-oven at 55 ºC for 48 h and urine was lyophilized. The dry matter (DM) of diets, orts, and feces was obtained by oven-drying at 102 ± 2 °C for 24 h (no. 934.01, AOAC [23]). Ash concentration (no. 942.05, AOAC [18]) and organic matter (OM) determination were attained by incineration of a sample in an electric muffle furnace at 550 ºC for 6 h. Feed, refusals, and feces were analyzed for neutral detergent fiber (aNDF) and acid detergent fibre (ADF) using the ANKOM Fiber Analyzer (A220, ANKOM Technologies, Fairport, NY, USA). The aNDF was NDF assayed with amylase (heat-stable) and expressed inclusive of residual ash. The lignin content was obtained by solubilization of cellulose with H_2_SO_4_, following procedures of Van Soest [24]. The sample was subjected to an acid hydrolysis before the ether extract (EE) was detached with petroleum ether (Soxhlet System HT Tecator, Hillerød, Denmark; 1047 Hydrolyzing Unit and 1043 Extraction Unit) using method no. 920.39 AOAC [23]. The Dumas principle (method no. 968.06, AOAC [23]) were used to determine the C and N amounts with the analyzer TruSpec CN; LECO Corporation, St. Joseph, MI, USA. The estimation of non-fibrous carbohydrate (NFC) of diets was obtained by difference, according to [25]. The gross energy (GE) content of the dried samples (feed, feces, and urine) was obtained by combustion in an adiabatic bomb calorimeter (Gallenkamp Autobomb; Loughborough, UK). 

Determination of VFA in ruminal liquor samples were described by Jouany [26] using a gas chromatograph (Fisons 8000 series; Fisons Instruments SpA, Milan, Italy) and the fatty acid (FA) methyl esters of orange leaf lipids were followed as described by O’Fallon et al. [27]. A Focus Gas Chromatograph (Thermo, Milan, Italy) was used to analyze FA methyl esters. Both equipment’s were armed with a split/splitless injector and a flame ionization detector. 

Metabolites in ruminal liquor, urine and blood plasma samples were analysed as follow. Briefly, glutamate and free amino groups and glutamate were determined according to Larsen [28]. Urine and plasma ammonium, total protein, urea, uric acid, albumin and glucose were analyzed agreeing to standard protocols (Siemens Diagnostics^®^ Clinical Methods for ADVIA 1800). Plasma non-esterified fatty acids (NEFA) were attained using the NEFA C ACS-ACOD assay method and, ß-hydroxy butyrate (BOHB) was realized as recommended by Harano et al. [29]. Both NEFA and BOHB analyses were performed on the ADVIA 1800 System. 

### 2.5. Calculations

The metabolizable energy (ME) intake (MEI) was the difference between intake and excretion. Therefore, the difference among GE intake (GEI) and energy losses in feces (E_feces_), urine (E_urine_) and CH_4_ (E_mehane_). The CH_4_ energy value was 39.5 kJ/L CH_4_, as reported by Brouwer [30].

Heat production (HP; in kJ) was calculated from indirect calorimetry recording the gas exchange (O_2_, CO_2_ and CH_4_; all gases measured in L), and urine N (N_urine_, g), using the Brouwer´s equation [30]:

HP (kJ) = 16.18 × O_2_ + 5.02 × CO_2_ − 2.17 × CH_4_ − 5.99 × N_urine._ The energy retention (RE) in the body was calculated as MEI − HP. The non-protein respiratory quotient (RQnp) was obtained following the next expression: RQnp = (CO_2_ − (N_urine_ × 6.25 × 0.774))/(O_2_ − (N_urine_ × 6.25 × 0.957)).

Efficiency of use of ME was determined according to [31]; km = 0.287 × q + 0.554), kf = 0.78 × q + 0.006, kmf = (km × kf × 1.5)/(kf +0.5 × km) with q being the energy metabolisability (the ratio ME/GE in the diet). The km was the ME efficiency for maintenance, kf the ME efficiency for growth and fattening, and kmf the ME efficiency for maintenance and growth. Although goats were not lactating and not pregnant, we considered a production level of 1.5 due to the fact they were fed beyond maintenance to reach a positive energy balance with the objective to enter mating when photoperiod was adequate. Digestible energy, ME, and NE of the diets were also calculated. From C and N balance the protein (R_protein_) and fat (R_fat_) retained in the body were calculated (g) following the method proposed by McLean [20].

### 2.6. Statistical Analysis 

Effects of replacement of barley straw and beet pulp with rice straw and orange leaves on intake, digestibility, ruminal fermentation, energy and C-N balances, and CH_4_ emission were studied using a mixed model (nlme library and lme function) in R [32]. 

The statistical model used was:

Y = μ + D + T + D × T + goat + ε

where Y was the dependent variable, μ was the overall mean, and D and T were the fixed effects of diet and period of time, and their interaction; goat was the random effect of goat; and ε was the random error. Least squares mean were obtained and differences were considered significant at *p* < 0.05.

## 3. Results 

No significant effect was observed for period of time and their interaction with diet in the crossover design; thus, the effect of diet was only reported in tables. The calibration factor from indirect calorimetry was 1.0043 ± 0.00126 (*n* = 4) for O_2_, 0.9951 ± 0.00982 (*n* = 4) for CO_2_ and 0.9655 ± 0.00623 (*n* = 4) for CH_4_. 

### 3.1. Feed Intake, Digestibility, and Ruminal Fermentation

A difference in DMI (*p* < 0.05) was observed between diets (0.34 kg/d, lower in CTR than ORG) (Table 2). Despite greater intake in the group of goats fed ORG, the apparent digestibility was lesser (*p* < 0.05) compared with CTR except for EE and CP, where no differences were observed. No differences were observed in EE digestibility between ORG and CTR, but ORG diet was richer in essential oils and polyunsaturated fatty acids (PUFA) due to the orange leaf (Table 3). 

Average ruminal pH never fell below 6.2 (Table 4), suggesting that values obtained were sufficiently high to maintain normal ruminal fermentation [33]. Although stomach tube is a suitable non-invasive technique for ruminal fluid sampling, it is prone to saliva contamination, which would increase the pH in the sample [34]. The NH_3_-N was higher in ORG than CTR and no differences were found for total VFA between diets (51.3 mM/L on average). However, differences were observed (*p* < 0.05) for propionic, isobutyric, and isovaleric acid. 

### 3.2. Energy Balance 

Due to the differences found between DMI, differences (*p* < 0.05) were found for GEI (1406 and 1654 kJ/kg of BW^0.75^ for CTR and ORG, respectively) (Table 5). Feces and urine energy losses were also bigger (*p* < 0.05) in response to feeding ORG compared with CTR. At the same time, this effect might partly explain the reduction (*p* < 0.05) in energy losses as CH_4_ (69 vs. 66 kJ/kg of BW^0.75^ for CTR and ORG, respectively). 

Due to greater losses in feces and urine with the ORG diet, the MEI was 199 kJ/kg of BW^0.75^ lower (*p* < 0.05) compared with CTR. Despite the differences in GEI between the diets, no significant differences were found for HP (482 kJ/kg of BW^0.75^, on average) and RE was positive with both treatments. Greater (*p* < 0.05) retention was detected in CTR compared with ORG (224 kJ/kg of BW^0.75^ greater in CRT than ORG). Change was not found for RQnp, with an averaged value of 1.01. 

Significant differences (*p* < 0.05) were observed for ME efficiencies. Efficiency of ME for maintenance is defined as km and, following the INRA [31] equations, a greater value was observed in response to feeding CTR compared with ORG (0.73 vs. 0.67 for CTR and ORG, respectively). The ME efficiency for growth and fattening (kf) according to INRA [31], was 0.47 and 0.31 for CTR and ORG, respectively. Because goats were in positive energy balance, we obtained the combined efficiency of maintenance, growth, and fattening (kmf); 0.61 and 0.48 was observed for CTR and ORG, respectively. 

When energy balance was expressed as percentage of GE intake, differences (*p* < 0.05) were found. Greater values were observed with CTR than ORG. The DEI/GEI was 18 points greater in CTR compared with ORG, and MEI/GEI was 21 points greater in CTR compared with ORG. When expressed per kg of DM, differences between diets (*p* < 0.05) were also detected for DE, ME, and NE. 

### 3.3. Carbon and Nitrogen Balance 

With the exception of C_CO2_, differences (*p* < 0.05) were found in C balance (Table 6). Following the trend observed for intake and apparent digestibility, C in feces and urine was 11.4 and 1.6 g/kg of BW^0.75^ greater with ORG compared with CTR, respectively. Losses in C from CH_4_ were lower with ORG compared with CON (0.03 g/kg of BW^0.75^), and total excretion of C was 14 g/kg of BW^0.75^ greater with ORG than CTR. With respect to N, goats consumed 0.8 g/kg of BW^0.75^ more with the ORG than CTR diet. However, excretion was 0.86 g/kg of BW^0.75^ greater with ORG than CTR. 

### 3.4. Metabolites

Ruminal, urine, and plasma metabolites primarily related to energy and protein metabolism did not differ greatly in response to diets, in part due to high variability (Table 7). Urea was greater with ORG than CTR in ruminal fluid and urine but non-significant, and difference (*p* < 0.05) was found in plasma. Uric acid in urine was higher (*p* < 0.05) in response to feeding ORG than CTR, and glutamate was higher (*p* < 0.05) with CTR than ORG. Greater (*p* < 0.05) branched-chain amino acid concentrations were observed in CTR than ORG and, no differences were detected for BOHB and NEFA.

### 3.5. Methane Emissions

The CH_4_ emission was shown in Table 8. Compared with the CTR diet, goats fed ORG emitted significantly (*p* < 0.05) fewer CH_4_ emissions (22.3 vs. 20.0 g/d for CTR and ORG, respectively). The Ym for both diets (CH_4_ conversion factor defined as E_methane_/GEI) was 4.9 and 4.0% with CTR and ORG, respectively (*p* < 0.05). In the current study, when CH_4_ emission was expressed over DM, OM intake, and fiber, statistical differences (*p* < 0.05) remained. 

## 4. Discussion

Higher intake in the ORG diet was accompanied by lower digestibility. One of the reasons for the reduction in digestibility could be the higher ash content (16 vs. 6%) in the ORG diet. Another reason was that EE in diet ORG was richer in PUFA, and could affect the digestibility of the fiber. Thus, PUFA could be responsible for the reduction in fiber digestibility. Classical work from Palmquist [35] indicated that dietary PUFA were more likely to inhibit fiber degradability with a concomitant reduction in fermentation, possibly due to coating food particles and preventing bacterial attachment. 

Average ruminal pH never fell below 6.2 suggesting that values obtained maintained normal ruminal fermentation [33]. The greater values of NH_3_-N in response to feeding ORG indicated poor utilization of dietary protein and extra N in the rumen. Regarding VFA, propionic acid was greater in response to feeding CTR and isobutyric and isovaleric were greater in goats fed ORG. As this VFA is mainly generated during degradation of branched-chain amino acids, the greater isovaleric acid concentration observed in goats fed ORG suggested greater ruminal protein degradation. The greater NH_3_-N in ORG diet could indicate an inefficient use of amino groups for ruminal protein synthesis [36]. Thus, the probable asynchrony between protein degradation and fiber carbohydrates appeared to be more pronounced with the ORG diet. 

Greater GEI was found in ORG than CTR due that the higher DMI observed in ORG. The smaller digestibility with the ORG diet indicated greater energy losses in feces, suggesting that the bigger high intake is accompanied by a higher rate of digesta transit. It could be possible that feeding ORG sustained the positive effect of lipids, essential oils, and tannins in the diet on CH_4_ reduction [9]. Due to greater energy losses with the ORG diet, the MEI was lower (640 vs. 839 kJ/kg of BW^0.75^ for ORG and CTR, respectively). The average value proposed by AFRC [37] using 17 estimates of ME for maintenance (MEm) derived from feeding trials with goats was 438 kJ/kg BW^0.75^ per day. Agreeing with other studies, the variation between estimates was substantial; ranging from 365 to 530 kJ/kg BW^0.75^ per day. Thus, Luo et al. [38] reviewing studies conducted with goats (dairy, meat, indigenous and Angora goats breeds, and several animal categories; dry and no pregnant, lactating goats, intact males, growing, and wethers) estimated MEm and it varied extensively; from 422 to 501 kJ/kg BW^0.75^ per day. The NRC [39] adopted for goats the maintenance requirements estimated from [38]. INRA [31] with a q of 0.64 suggested 441 kJ/kg BW^0.75^ per day. Prieto et al. [40] working with adult and castrated male goats attained a value of 443 kJ MEm/kg BW^0.75^ per day, and Aguilera et al. [41] obtained 401 kJ/kg BW^0.75^ per day. The model obtained by Fernández [42] had values of 460 kJ MEm/kg BW^0.75^ per day. Averaging the studies mentioned above, the MEm would be 445 kJ/kg BW^0.75^ per day. As the MEI obtained was 839 and 640 kJ/kg BW^0.75^ per day for the CTR and ORG diet, respectively, diet CTR had more energy available for weight recovery (395 and 196 kJ ME/kg BW^0.75^ per day for CTR and ORG, respectively). The value of ME expressed over kg of DM was 11 and 6 MJ ME/kg DM in response to feeding CTR and ORG, respectively. Therefore, the RE was 370 kJ/kg BW^0.75^ for CTR and 146 kJ/kg BW^0.75^ for ORG, suggesting that poor microbial protein synthesis coupled with lower content of glucogenic nutrients with the ORG diet (i.e., CTR diet contain beet pulp) did not favor the partitioning of ME into body tissue and, hence, the body fat deposition [43].

Maintenance costs represent the main component of total energy requirements, which underscores the importance for an accurate estimation. The km obtained was 0.73 vs. 0.67 for CTR and ORG, respectively. The AFRC [37] and NRC [39] systems for goats estimated ME efficiency for maintenance using the same equation, which varies from 0.64 (low-quality diets) to 0.75 (high-quality diets). Using indirect calorimetry and regression techniques, Prieto et al. [40] obtained a value of 0.73 with adult Granadina castrated males’ goats and Aguilera et al. [41] obtained a value of 0.67 with lactating Granadina goats. Thus, CTR appears to have been a better-quality diet with the km obtained and the effectiveness found being within the ranges that literature reported. The kmf was 0.61 and 0.48 for CTR and ORG, respectively. If calculating a ratio between RE and MEI from Table 5, the values obtained were 0.44 and 0.23 for CTR and ORG, respectively. The ME efficiency for maintenance and recovery of body reserves followed the same trend, and was greater in CTR than ORG. The ME requirements for body gain differs due to changes from ME to RE, depending on even if energy derived from feed (lipogenic or glucogenic nutrient as Van Knegsel [43] reported) or body fat mobilization [44]. Thus, with both diets, animals recovered energy despite the fact that the effectiveness of the diet was greater for CTR.

Efficiency of C retained over C ingested was 23% and 8% when feeding CTR and ORG, respectively. In another study with Murciano-Granadina dry and non-pregnant goats, feeding close to maintenance the value obtained was 5% [17]. Another study with lactating Muciano-Granadina goats generated a value ranging from 4% to 9% [45]. The ratio between retained N and ingested N was again lower when feeding ORG than CTR (19% vs. 10% for CTR and ORG, respectively), indicating that the amount of protein in the ORG diet was above the animal’s needs. In the study of Fernández et al. [17], the value obtained was 38%, and Romero et al. [45] reported values ranging from 12% to 15%. From the C and N retained, retention of protein and fat were estimated [20]. Differences were detected between diets and the R_protein_ was 9 g/goat greater in response to feeding CTR than ORG. The R_fat_ was approximately 108 g/goat greater with CTR than ORG. 

Urea was greater with ORG than CTR in plasma, indicating lower feed protein efficiency [46]. Urine uric acid concentration was higher in response to feeding ORG than CTR, again indicating that feeding ORG led to poor microbial protein synthesis and greater excretion of N compounds. The higher glutamate with CTR than ORG likely contributed to glutamine synthesis across the animal tissues [28]. Branched-chain amino acids may be used for synthesis of muscle or milk protein, preserved into the cell for structural protein synthesis, used as precursor for different metabolic and catabolic processes, or passes unaltered into milk, blood, or lymph [47]. The greater branched-chain amino acid concentrations observed in response to feeding CTR than ORG could be indicative of greater utilization by other peripheral tissues such as adipose [48]. Additionally, the lack of differences for BOHB or NEFA suggested little effect of diets in whole-body energy balance [49]. 

The reduction in CH_4_ when feeding ORG was partly caused by decreasing overall carbohydrate digestion. Goats fed ORG emitted 2.3 g/d less CH_4_ than CTR, a response that agrees with the fact that FA from orange leaves have inhibitory effect on protozoa and cellulolytic bacteria that can cause shifts in fermentation patterns that reduce CH_4_ production [3,11]. In addition to the known negative effects of PUFA on CH_4_ production via direct toxic effects on ruminal microorganism and protozoa [50,51], secondary metabolites compounds in plants like tannins and essential oils from orange leaves in the ORG diet also could explain the mitigation of CH_4_ observed with this diet [9,52,53]. Thus, although the reduction in CH_4_ averaged 10%, differences in fat percentage between CTR and ORG averaged 1.1%. Because ruminants waste between 2 and 12% of their dietary GEI as CH_4_, a diminution in CH_4_ production represents an improvement in feed efficiency [54]. The Ym was 4.9 and 4.0% with CTR and ORG, respectively. Concerning the ORG diet, the FA profile of ORG (Table 3) probably had a negative effect on methanogens and fiber degradation, which was reflected in the lower NDF and ADF digestibility (Table 2). While CH_4_ emissions were most commonly expressed in the literature over GEI, the other meaningful indicator was over DM or OM intakes. The CH_4_ reduction was observed also when it was expressed relative to DM, OM, and different fiber fractions. Patra et al. [55] reported that some plant secondary metabolites such as saponins and tannins, which are present in orange leaves, may show an inhibitory effect on methanogenic activity. Thus, further research should be performed to better understand how secondary compounds in citrus residue directly impact ruminal microorganisms.

## 5. Conclusions

The present study provides data for energy, C-N balance, and CH_4_ emissions in Murciano-Granadina goats to support maintenance when crops and pruning waste by-products were fed. The diet replaced barley straw and beet pulp with rice straw and orange leaves, keeping sunflower meal as the same source of protein. Despite the grater feed intake when supplementing orange leaves and rice straw, the digestibility and energy balance were lower. The MEI was 199 kJ/kg of BW^0.75^ lower in response to feeding orange leaves and the value of ME was 6 and 11 MJ ME/kg DM for that diet and the control, respectively. Retention of energy was 224 kJ/kg of BW^0.75^ greater with the control and positive with both diets. Greater efficiency of maintenance, growth, and fattening was detected when feeding the control (0.61) compared with orange leaves diet (0.48). The efficiency of C retained over C ingested was 15 points greater with the control diet, and the N efficiency 9 points greater with the control diet. Reduction in CH_4_ emissions were detected with the orange diet (from 22.3 to 20.0 g/d). Thus, despite the lower energy, C and N balance efficiencies when orange leaves were supplemented, goats recovered body reserves, and, consequently, these fibrous by-products were utilized without detectable unfavorable effects on energy metabolism. The economic advantages and environmental impact of feeding orange leaves should be assessed. 

## Figures and Tables

**Table 1:** Ingredients and chemical composition of diets.

Item	Diet ^1^	
	CTR	ORG
*Ingredients, % DM*		
barley straw	27	0
rice straw	0	24
beet pulp	45	0
orange leaves	0	45
sunflower meal 28	20	23
sugarcane molasses	7	7
calcium carbonate	0.7	0.5
sodium chloride	0.3	0.3
*Chemical composition, % of DM*		
Dry matter	96	96
Organic matter	90	80
Ash	6	16
Crude Protein	12	14
Ether rextract	1.1	1.1
Neutral detergent fiber	50	44
Acid detergent fiber	31	29
Acid detergent lignin	5	7
Hemicellulose	19	15
Cellulose	26	23
NFC ^2^	31	26
Carbon	45	42
Nitrogen	2	2
Gross energy, MJ/kg DM ^3^	17	16

^1^ CTR = control; ORG = orange leaves and rice straw. ^2^ NFC = non fibrous carbohydrate; 100 − (NDF + ash + CP + EE). ^3^ DM = Dry matter.

**Table 2:** Body weight, feed intake, and apparent total tract digestibility (% of DM) of Murciano-Granadina dried goats (*n* = 10) by the type of diet.

Item ^1^	Diet ^2^		SEM ^3^	*p*-Value
	CTR	ORG		
BW	46.6	41.5	0.94	0.0049
DMI, kg/d	1.41	1.74	0.273	0.0001
DM	67	46	2.1	0.0001
OM	69	51	1.8	0.0001
EE	45	46	1.4	0.7261
CP	59	62	0.9	0.2073
NDF	59	37	2.3	0.0001
ADF	58	36	2.3	0.0001
ADL	25	21	1.9	0.2710
hemicellulose	62	40	2.3	0.0001
cellulose	64	41	2.4	0.0001
NFC	89	67	1.9	0.0001
GE	68	50	1.8	0.0001

^1^ BW = body weight; BW^0.75^ = metabolic body weight; DMI = dry matter intake; DM = dry matter; OM = organic matter; CP = crude protein; EE = ether extract; NDF = neutral detergent fiber; ADF = acid detergent fiber; NFC = non fibrous carbohydrate: 100 − (NDF + ash + CP + EE); GE = gross energy. ^2^ CTR = control; ORG = orange leaves and rice straw. ^3^ SEM = Standard error of the mean.

**Table 3:** Fatty acid profile from orange leaves (mg/100 mg).

Fatty Acids	Orange Leaves
C4:0	0.000
C6:0	0.000
C8:0	0.000
C10:0	0.001
C11:0	0.000
C12:0	0.017
C13:0	0.122
C14:0	0.034
C14:1	0.000
C15:0	0.002
C16:0	0.274
C16:1	0.001
C17:0	0.010
C17:1	0.000
C18:0	0.039
C18:1n9t	0.000
C18:1n9c	0.057
C18:1n7	0.010
C18:2n6t	0.000
C18:2n6c	0.326
C20:0	0.013
C18:3n6	0.006
C20:1	0.000
C18:3n3	0.294
^1^ CLA 9c11t + 9t11c	0.000
C20:2	0.000
C22:0	0.018
C20:3n6	0.014
C22:1n9	0.004
C20:3n3	0.008
C24:0	0.022

^1^ CLA = conjugated linoleic acid.

**Table 4:** Ruminal parameters: pH, NH_3_-N, and volatile fatty acids (VFA) of Murciano-Granadina dried goats (*n* = 10) by the type of diet.

Item ^1^	Diet ^2^		SEM ^3^	*p*-Value
	CTR	ORG		
pH	6.5	6.9	0.05	0.2155
NH_3_-N, mg/dL	12.3	15.1	0.44	0.0125
Total VFA, mM/L	54.2	48.4	3.11	0.6212
*Individual VFA, mM/L*				
Acetic acid	32.7	31.9	1.73	0.8214
Propionic acid	11.8	8.1	0.87	0.0245
iso Butyric acid	0.30	0.66	0.065	0.0001
Butyric acid	8.30	5.93	0.748	0.1169
Isovaleric acid	0.24	0.79	0.103	0.0004
n-Valeric acid	0.79	0.90	0.058	0.3376
n-Caproic acid	0.08	0.10	0.012	0.5665
Heptanoic acid	0.000	0.002	0.0008	0.1479

^1^ NH_3_-N = ammonia nitrogen. ^2^ CTR = control; ORG = orange leaves and rice straw. ^3^ SEM = Standard error of the mean.

**Table 5:** Energy balance (kJ/kg of BW^0.75^ and day) of Murciano-Granadina dried goats (*n* = 10) by the type of diet.

Item ^1^	Diet ^2^		SEM ^3^	*p*-Value
	CTR	ORG		
GEI	1406	1654	46.4	0.0060
E_feces_	447	824	38.5	0.0001
E_urine_	51	124	7.4	0.0001
DEI	959	830	31.3	0.0385
E_methane_	69	66	2.0	0.0224
MEI	839	640	36.0	0.0043
HP	469	494	8.8	0.1653
RE	370	146	36.5	0.0013
RQnp	1.03	0.99	0.015	0.1254
*ME efficiencies*				
km	0.73	0.67	0.014	0.0302
kf	0.47	0.31	0.012	0.0001
kmf	0.69	0.45	0.011	0.0001
% GEI				
DEI	68	50	2.8	0.0001
MEI	60	39	2.3	0.0001
RE	26	9	2.6	0.0001
*Diet energy value, MJ/kg DM*				
DE	12	8	0.3	0.0001
ME	11	6	0.1	0.0001

^1^ GEI = gross energy intake; E_feces_ = energy losses in feces; E_urine_ = energy losses in urine; E_methane_ = energy losses in methane; DEI = digestble eible nergy intake; ME = metabolizable energy; MEI = metabolizable energy intake; HP = heat production; RE = energy retention; RQnp = respiration quotient corrected to nitrogen; km = ME efficiency for maintenance; kf = ME efficiency for fattening; kmf = ME efficiencies for maintenance and fattening. ^2^ CTR = control; ORG = orange leaves and rice straw. ^3^ SEM = standard error of the mean.

**Table 6:** Daily balance of carbon and nitrogen (g/kg de BW ^0.75^) of Murciano-Granadina dried goats (*n* = 10) by the type of diet.

Item ^1^	Diet ^2^		SEM ^3^	*p*-Value
	CTR	ORG		
C_intake_	35.69	45.13	1.296	0.0001
C_feces_	11.93	23.29	1.129	0.0001
C_urine_	1.21	2.85	0.172	0.0001
C_CO2_	13.35	14.41	0.258	0.1383
C_CH4_	0.94	0.91	0.008	0.0256
C excretion	27.44	41.46	1.389	0.0001
C_retained body_	8.25	3.67	0.555	0.0001
N_intake_	1.56	2.36	0.085	0.0001
N_feces_	0.64	0.90	0.032	0.0001
N_urine_	0.63	1.22	0.085	0.0001
N excretion	1.27	2.12	0.100	0.0001
N_retained body_	0.30	0.24	0.067	0.0123
R_protein_, g/goat	33	25	3.3	0.0158
R_fat_, g/goat	169	62	12.6	0.0024
Expected gain, g/d	302	160	21.4	0.0001

^1^ C = carbon; N = nitrogen; R = retention. ^2^ CTR = control; ORG = orange leaves and rice straw. ^3^ SEM = standard error of the mean.

**Table 7:** Metabolites in rumen liquor, urine, and plasma of Murciano-Granadina dried goats (*n* = 10) by the type of diet.

Item	Diet ^1^			*p*-Value
	CTR	ORG	SEM ^2^	
*Rumen liquor*				
Free amino groups, mEq/L	1.28	1.02	0.099	0.2121
Glutamate, mM	117	107	9.1	0.5945
Urea, mM	27.8	33.1	3.49	0.4826
*Urine*				
Protein, mg/L	304	215	48.0	0.3844
Free amino groups, mEq/L	13.7	17.0	1.99	0.4349
Glutamate, mM/L	74	41	6.4	0.0024
Urea, mM/L	601	621	44.6	0.8345
Uric acid, mM/L	224	471	80.8	0.0452
Malate, mM/L	21.8	28.2	2.81	0.2787
*Plasma*				
Protein, g/L	81	81	1.8	0.9836
Albumin, g/L	35	36	0.8	0.8414
Glutamate, µM/L	65	49	4.9	0.0351
Free amino groups, mEq/L	2.8	2.4	0.3	0.5040
Free Branched Chain Amino acids, mM/L	0.78	0.56	0.056	0.0423
Urea, mM/L	5.8	7.3	0.29	0.0020
Glucose, mM/L	4.0	4.0	0.21	0.9449
BOHB ^3^, mM/L	0.34	0.31	0.026	0.5431
NEFA ^4^, mEq/L	111	159	23.4	0.3328

^1^ CTR = control; ORG = orange leaves and rice straw. ^2^ SEM = standard error of the mean. ^3^ BOHB = Beta-Hydroxybutyrate. ^4^ NEFA = nonesterified fatty acids.

**Table 8:** Methane emission of Murciano-Granadina dried goats (*n* = 10) by the type of diet.

Item ^1^	Diet ^2^		SEM ^3^	*p*-Value
	CTR	ORG		
CH_4_, g/d	22.3	20.0	0.482	0.035
ratio CH_4_/CO_2_ in breath	0.07	0.06	0.001	0.0070
Ym	4.9	4.0	0.07	0.0018
CH_4_/DMi, g/kg	16.0	11.6	0.577	0.0001
CH_4_/OMi, g/kg	17.1	13.8	0.564	0.0025
CH_4_/NDFi, g/kg	32.4	26.7	1.055	0.0010
CH_4_/ADFi, g/kg	52.1	40.0	1.788	0.0003
CH_4_/hemicellulose intake, g/kg	3.0	1.7	0.134	0.0001
CH_4_/cellulose intake, g/kg	61.9	51.6	1.998	0.0089

^1^ Ym = methane energy/gross energy intake; DMi = dry matter intake; OMi = organic matter intake; NDFi = neutral detergent fiber intake; ADFi = acid detergent fiber intake. ^2^ CTR = control; ORG = orange leaves and rice straw. ^3^ SEM = standard error of the mean.

## Data Availability

All data used in the current study are available from the corresponding author on reasonable request.

## References

[1] Salami S.A., Luciano G., O’Grady M.N., Biondi L., Newbold C.J., Kerry J.P., Priolo A. (2019). Sustainability of feeding plant by-products: A review of the implications for ruminant meat production. Anim. Feed Sci. Technol..

[2] Ajila C., Brar S., Verma M., Tygi R., Godbout S., Valéro J. (2012). Bio-processing of abro-byproducts to animal feed. Crit. Rev. Biotechnol..

[3] Leng R.A. (1993). Quantitative ruminant nutrition: A green science. Aust. J. Agric. Res..

[4] Van Soest P.J. (2006). Rice straw, the role of silica and treatments to improve quality. Anim. Feed Sci. Technol..

[5] FAOSTAT FAO Statistical Data Base Food and Agricultural Organization If the United Nations. http//faostat.fao.org.

[6] EUROSTAT European Commission Data Base. http://ec.europa.eu/Eurostat/data/database.

[7] EFEAGRO EFE Agency for the Agrifood Sector, 28036 Madrid, Spain. http://efeagro.com.

[8] Bampidis V.A., Robinson P.H. (2006). Citrus by-products as ruminant feeds: A review. Anim. Feed Sci. Technol..

[9] Knapp J.R., Laur G.L., Vadas P.A., Weis W.P., Tricarico J.M. (2014). Invited review: Enteric methane in dairy cattle production: Quantifying the opportunities and impact of reducing emissions. J. Dairy Sci..

[10] Alzawqari M.H., Al-Baddany A.A., Al-Baadani H.H., Alhidary I.A., Ullah Khan R., Aqil G.M., Abdurab A. (2016). Effect of feeding dried sweet orange (*Citrus sinensis*) peel and lemon grass (*Cymbopogon citratus*) leaves on growth performance, carcass traits, serum metabolites and antioxidant status in broiler during the finisher phase. Environ. Sci. Pollut. Res. Int..

[11] Fernández C., Pérez-Baena I., Martí J.V., Palomares J.L., Jorro-Ripoll J., Segarra J.V. (2019). Use of orange leaves as a replacement for alfalfa in energy and nitrogen partitioning, methane emissions and milk performance of Murciano-Granadina goats. Anim. Feed Sci. Technol..

[12] Wei M., Cui Z.-H., Li J.-W., Yan P.-S. (2018). Estimation of metabolisable energy and net energy of rice straw and wheat straw for beet cattle by indirect calorimetry. Arch. Anim. Nutr..

[13] Nazli M.H., Halim R.A., Abdullah A.M., Hussin G., Samsudin A.A. (2018). Potential of feeding beef cattle with whole corn crop silage and rice straw in Malaysia. Trop. Anim. Health Prod..

[14] Quang D.V., Ba N.X., Doyle P.T., Hai D.V., Lane P.A., Malau-Aduli A.E., Van N.H., Parsons D. (2015). Effect of concentrate supplementation on nutrient digestibility and growth of Brahman crossbred cattle fed a basal diet of grass and rice straw. J. Anim. Sci. Technol..

[15] European Union (2007). European Directive 86/609/EEC of 24 November 1986. Commission Recommendation of 18 June 2007 on Guidelines for the Accommodation and Care of Animals Used for Experimental and Other Scientific Purposes. Annex II to European Council Directive 86/609/EEC.

[16] Calsamiglia S., Bach A., de Blas C., Fernández C., García-Rebollar P. (2009). Nutritional Requirements for Dairy Ruminants.

[17] Fernández C., López M.C., Lachica M. (2012). Description and function of a mobile open-circuit respirometry system to measure gas exchange in small ruminants. Anim. Feed Sci. Technol..

[18] Fernández C., López M.C., Lachica M. (2015). Low cost open-circuit hood system for measuring gas exchange in small ruminants: From manual to automatic recording. J. Agric. Sci..

[19] Fernández C., Gomis-Tena J., Hernández A., Saiz J. (2019). An open circuit indirect calorimetry head hood system for measuring methane emissions and energy metabolism in small ruminants. Animals.

[20] McLean J.A., Tobin G. (1987). Animal and Human Calorimetry.

[21] Brockway J.M., Boyne A.W., Gordon J.G. (1971). Simultaneous calibration of gas analyzers and meters. J. Appl. Physiol..

[22] Aguilera J.F., Prieto C. (1986). Description and function of an open-circuit respiration plant for pigs and small ruminants and the techniques used to measure energy metabolism. Arch. Anim. Nutr..

[23] AOAC International (2000). Official Methods of Analysis of the Association of Official Analytical Chemists.

[24] Van Soest P.J., Wine R.H. (1968). Determination of lignin and cellulose in acid detergent fiber with permanganate. J. Assoc. Off. Anal. Chem..

[25] National Research Council (NRC) (2001). Nutrient Requirements of Dairy Cattle.

[26] Jouany J.P. (1982). Volatile fatty acid and alcohol determination in digestive contents, silage juices, bacterial cultures and anaerobic fermentor contents. Sci. Aliments.

[27] O’Fallon J.V., Busboom J.R., Nelson M.L., Gaskins C.T. (2007). A direct method for fatty acid methyl ester synthesis: Application to wet meat tissues, oils, and feedstuffs. J. Anim. Sci..

[28] Larsen T., Fernandéz C. (2017). Enzymatic-fluorometric analyses for glutamine, glutamate and free amino groups in protein-free plasma and milk. J. Dairy Res..

[29] Harano Y., Ohtsuki M., Ida M., Kojima H., Harada M., Okanishi T., Kashiwagi A., Ochi Y., Uno S., Shigeta Y. (1985). Direct automated assay method for serum or urine levels of ketone bodies. Clin. Chim. Acta..

[30] Brouwer E., Blaxter K.L. (1965). Report of sub-committee on constants and factors. Proceedings of the 3rd Symposium on Energy Metabolism.

[31] (2018). INRA Feeding System for Ruminants.

[32] R Core Team (2016). R: A Language and Environment for Statistical Computing.

[33] Ørskov E.R., Fraser C. (1975). The effects of processing of barley –based supplements on rumen pH, rate of digestion and voluntary intake of dried grass in sheep. Br. J. Nutr..

[34] Ramos-Morales E., Arco-Pérez A., Martín-García A.I., Yáñez-Ruiz D.R., Frutos P., Hervás G. (2014). Use of stomach tubing as an alternative to rumen cannulation to study ruminal fermentation and microbiota in sheep and goats. Anim. Feed Sci. Technol..

[35] Palmquist D.L., Jenkins T.C. (1980). Fat in lactation rations: Review. J. Dairy Sci..

[36] Casper D.P., Maiga H.A., Brouk M.J., Schingoethe D.J. (1999). Synchronization of carbohydrate and protein sources on fermentation and passage rates in dairy cows. J. Dairy Sci..

[37] Agricultural and Food Research Council (AFRC) (1993). Energy and Protein Requirements of Ruminants.

[38] Luo J., Goetsch A.L., Nsahlai I.V., Johnson Z.B., Sahlu T., Moore J.E., Ferrell C.L., Galyean M.L., Owens F.N. (2004). Maintenance energy requirements of goats: Predictions based on observations of heat and recovered energy. Small Rum. Res..

[39] National Research Council (NRC) (2007). Nutrient Requirements of Small Ruminants. Sheep, Goats, Cervids and New World Camelids.

[40] Prieto C., Aguilera J.F., Lara L., Fonollá J. (1990). Protein and energy requirements for maintenance of indigenous Granadina goats. Brit. J. Nutr..

[41] Aguilera J.F., Prieto C., Fonollá J. (1990). Protein and energy metabolism of lactating Granadina goats. Br. J. Nutr..

[42] Fernández C., Romero T. (2019). Energy Balance Data from Lactating Dairy Goats Offered Total Mixed Diets. Open J. Anim. Sci..

[43] Van Knegsel A.T.M., van den Brand H., Dijkstra J., van Straalen W.M., Heetkamp M.J., Tamminga S., Kemp B. (2007). Dietary energy source in dairy cows in early lactation: Energy partitioning and milk composition. J. Dairy Sci..

[44] Moraes L.E., Kebreab E., Strathe A.B., Dijkstra J., France J., Casper D.P., Fadel J.G. (2015). Multivariate and univariate analysis of energy balance data from lactating dairy cows. J. Dairy Sci..

[45] Romero T., Pérez-Baena I., Larsen T., Gomis-Tena J., Loor J.J., Fernández C. (2020). Inclusion of lemon leaves and rice straw into compound feed and its effect on nutrient balance, milk yield, and methane emissions in dairy goats. J. Dairy Res..

[46] Spek J.W., Dijkstra J., Van Duinkerken G., Bannink A. (2013). A review of factors influencing milk urea concentration and its relationship with urinary urea excretion in lactating dairy cattle. J. Agric. Sci..

[47] Larsen M., Galindo C., Ouellet D.R., Maxin G., Kristensen N.B., Lapierre H. (2015). Abomasal amino acid infusion in postpartum dairy cows: Effect on whole-body, splanchnic, and mammary amino acid metabolism. J. Dairy Sci..

[48] Leal Yepes F.A., Mann S., Overton T.R., Ryan C.M., Bristol L.S., Granados G.E., Nydam D.V., Waklhlag J.J. (2018). Effect of rumen protected branched-chain amino acid supplementation on production and energy related metabolites during the first 35 days in milk Holstein dairy cows. J. Dairy Sci..

[49] Bjerre-HarpØth V., Friggens N.C., Thorup V.M., Larsen T., Damgaard B.M., Ingvartsen K.L., Moyes K.M. (2012). Metabolic and production profiles of dairy cows in response to decreased nutrient density to increase physiological imbalance at different stages of lactation. J. Dairy Sci..

[50] Newbold C.J., Lassalas B., Jouany J.P. (1995). The importance of methanogens associated with ciliated protozoa in ruminal methane production in vitro. Lett. Appl. Microbiol..

[51] Patra A. (2014). A meta-analysis of the effect of dietary fat on enteric methane production, digestibility and rumen fermentation in sheep, and a comparison of these responses between cattle and sheep. Livest. Sci..

[52] Patra A., Yu Z. (2012). Effect of essential oils on methane production and fermentation by, and abundance and diversity of, rumen microbial populations. Appl. Environ. Microbiol..

[53] Calsamiglia S., Busquet M., Cardozo P.W., Castillejos L., Ferret A. (2007). Invited Review: Essential oils as modifiers of rumen microbial fermentation. J. Dairy Sci..

[54] Johnson K.A., Johnson D.E. (1995). Methane emissions in cattle. J. Anim. Sci..

[55] Patra A., Park T., Minseok K., Yu Z. (2017). Rumen methanogens and mitigation of methane emission by anti-methanogenic compounds and substances. J. Anim. Sci. Biotech..

